# Where is critical analysis of power and positionality in knowledge translation?

**DOI:** 10.1186/s12961-021-00726-w

**Published:** 2021-06-11

**Authors:** Chloe Crosschild, Ngoc Huynh, Ismalia De Sousa, Eunice Bawafaa, Helen Brown

**Affiliations:** 1 Faculty of Applied Science, School of Nursing, University of British Columbia, T201-2211 Wesbrook Mall, Vancouver, BC V6T 2B5 Canada; 2 School of Nursing, University of Northern British Columbia, 3333 University Way, Prince George, BC V2N 4Z9 Canada

**Keywords:** Knowledge translation, Integrated knowledge translation, Power dynamics, Knowledge user, Critical reflexivity, Relationality, Critical theory, Black feminist thought, Indigenous knowledge

## Abstract

In Canada, the Eurocentric epistemological foundations of knowledge translation (KT) approaches and practices have been significantly influenced by the Canadian Institutes of Health Research (CIHR) KT definition. More recently, integrated knowledge translation (IKT) has emerged in part as epistemic resistance to Eurocentric discourse to critically analyse power relations between researcher and participants. Yet, despite the proliferation of IKT literature, issues of power in research relationships and strategies to equalize relationships remain largely unaddressed. In this paper, we analyse the gaps in current IKT theorizing against the backdrop of the CIHR KT definition by drawing on critical scholars, specifically those writing about standpoint theory and critical reflexivity, to advance IKT practice that worked to surface and change research-based power dynamics within the context of health research systems and policy.

## Background

The gold standard for developing reputable healthcare research has been grounded in empiricism and Western science [1]. The rise of the evidence-based movement throughout the 1980s furthered the status of empiricism and Western science as the defensible method to produce rigorous research outcomes [2]. Initially intended for medical practitioners as a way to prepare, maintain, and disseminate systematic reviews into practice, the evidence-based practice was also driven by the long-standing aim to *close* the knowledge–action gap [2]. Within Western evidence paradigm derived from empiricism, “evidence” reflects positivist assumptions about the nature of knowledge [3] that, in turn, become problematic and ideological when considering the ways in which positivism is built upon the norms of Western thought that reify cognitive imperialism [4, 5]. Evidence-based practice as the basis for knowledge translation (KT) approaches within healthcare practice, policy, and research contributes to exclusionary, normative, and ideological forms of knowing—predicated on Western rationalization—that simultaneously exclude other forms of and claims about what constitutes knowledge [1, 2, 6–8].

To advance the dissemination and uptake of empirically derived knowledge in health policy and practice, KT emerged as a field of study to advance the implementation of research-derived evidence within a complex system of exchange among stakeholders [2]. Early KT discourse contributed to expanding what constitutes evidence, offering a more robust framework for developing knowledge where ontological and epistemological diversity would find space and support [9]. While expanded discourse on evidence has emerged within the literature, many argue that the knowledge–action gap persists in health research and policy [10, 11], while others problematized the very nature and existence of this “gap” itself [8]. What receives less attention in KT scholarship is how ontological and epistemological orientations of researchers are ideologically rooted in Western Eurocentrism, such that non-Western Eurocentric KT approaches run the risk of being rejected and erased in favour of more “scientific” evidence rooted in rationalization and the modern ontology [12, 13]. As emerging nursing scholars who are currently enrolled in doctoral education, we argue in this paper for critical analysis of Eurocentric epistemological foundations of existing KT approaches to contribute to *evolving* and *expanding* KT discourse and frameworks wherein power and positionally are adequately theorized within knowledge hierarchies, and voices and perspectives of research participants are incorporated into research frameworks. Within the context of contemporary KT discourse, we join researchers taking on integrated knowledge translation (IKT) which is “…rooted in and committed to prioritizing researcher–community relationships and creating an ethical space for different forms of knowledge” [14], and discuss the need for explicit dialogue about the ethical spaces of IKT, rooted in more critical analysis of power relations, knowledge hierarchies, and research–participant standpoints. We consider this space an entry point for *expanding* critical theorizing necessary to advance IKT for nursing and healthcare research, policy, and services. We begin by problematizing the erasure of power and positionality within the KT literature. We then examine the uptake of IKT in nursing and health research by surfacing the discursive construction of power in Western academic institutions and health systems. We then shift to proposing how IKT scholarship and practice can be advanced through critical theoretical analysis and action with explicit attention to (1) deconstructing language to render inherent power differentials visible among academic institutions, researchers, and knowledge partners; (2) surfacing how IKT can advance through dialogue and practices founded on epistemic pluralism and social justice; and (3) interrogating knowledge hierarchies through critical reflexivity as the basis for equitable researcher–participant relationships.

## CIHR knowledge translation definition

The Canadian Institutes of Health Research (CIHR) definition of KT [6] is the most widely used definition in health research within Canada [15] and draws from the work of Graham [16, 17]:

A dynamic and iterative process that includes synthesis, dissemination, exchange and ethically-sound application of knowledge to improve the health of Canadians, provide more effective health services and products and strengthen the health care system. This process takes place within a complex system of interactions between researchers and knowledge users which may vary in intensity, complexity and level of engagement depending on the nature of the research and the findings as well as the needs of the particular knowledge user [18].

This definition amplifies neoliberal ideologies driven by individualistic and finance-driven values [6, 19–21]. Specifically, the “exchange” of knowledge assumes a transaction between individuals, rather than holistic approaches to knowing, being, and living. Moreover, knowledge “exchange” reflects self-consumerism and a market-oriented economy and assumes the superiority of Western and Eurocentric values over those of non-Western, non-European societies [5, 22]. Similarly, the CIHR KT definition emphasizes the complexities and variability of KT and uses specific language to identify the various positions people occupy within the knowledge-to-action cycle (KTA). For example, a *knowledge user* is defined as:

An individual who is likely to be able to use the knowledge generated through research to make informed decisions about health policies, programs and/or practices. A knowledge user’s level of engagement in the research process may vary in intensity and complexity depending on the nature of the research and his/her information needs. A knowledge-user can be but is not limited to, a practitioner, policy-maker, educator, decision-maker, health care administrator, community leader, or an individual in a health charity, patient group, private-sector organization, or media outlet. —Canadian Institutes of Health Research [23].

The CIHR definition acknowledges the “complex system of interactions” within research, pointing to features of those interactions as being “intense”, reflecting the level of engagement, nature of the research, and the needs of the knowledge user. In our discussion that follows, we argue that the framing of key partners and stakeholders as “knowledge users” emphasizes a functional role in the uptake of research that oversimplifies the complex interactions that are inherent within research relationships imbued with power and positionality and reflect Eurocentrism and Western knowledge paradigms within dominant KT discourse.

### Knowledge users: Eurocentrism and Western knowledge paradigms

The CIHR definition for *knowledge user* signifies researchers as the sole producers of knowledge, whereas stakeholders and research partners within policy, practice, and healthcare contexts are positioned for effective “use” or uptake. The language of “user” reflects Western knowledge paradigms where knowledge and action/uptake are considered distinct KT activities within KTA [18]. Being users of knowledge, partners, participants, and communities are ideologically positioned within a specific role within the KTA to “use” knowledge, rendering the possibility of knowledge co-creation and participatory approaches less visible within KT [2]. It simultaneously implies that stakeholders’, decision-makers’, partners’, and communities’ primary role is to *use* the knowledge provided to them by researchers [2]. We argue this KTA framing and language of the CIHR KT definition acts ideologically to reify the power imbalances that permeate Western academic institutions that ultimately undermine KT outcomes. The expertise and ability of individuals and communities to contribute to the process of knowledge creation is rendered less visible for the effective “uptake” of research for change or action in healthcare practice or policy [15]. Language use and its impacts on research acts discursively to produce epistemic privilege within Eurocentric Western knowledge paradigms where non-researchers are constructed as secondary knowledge producers [20]. Normative discourse constitutes praxis in academic structures, reflecting power relations and hierarchy that privilege the ontological and epistemological views of the researcher [5, 24, 25].

The evolution of integrated KT (IKT) scholarship and practice in nursing and healthcare is turning attention towards the place of pragmatism, epistemological pluralism, and social justice and equity [26, 27] as relevant considerations for effective KTA. Pragmatism focuses on how the meaning of phenomena can only be validated by evaluating their practical consequences in terms of actions [26, 28]. Pragmatism rests on the claim that all knowledge/knowing is inherently linked to practice [9, 26]; IKT is helping to shift normative Western discourse about the binary construction of knowledge and action as distinct, whereby researchers create knowledge and then delegate its actionable uptake into policy and practice to stakeholders [16].

Within this space, three points of tension surface from the KTA model in the context of nursing and healthcare that are worthy of epistemic resistance. Firstly, by using the terminology “knowledge creation”, the KTA model is based on assumptions that all knowledge is created; this underlying assumption contradicts many Indigenous ways of knowing wherein there is no new creation of knowledge, but rather a “process of gradual awareness and understanding of complex, interconnected, and pluralistic systems of existing knowledge” [21, 29, 30]. Within Indigenous knowledge systems, knowledge is neither individually created nor static; an underlying assumption within diverse Indigenous cosmologies is that knowledge emerges out of relationships with the land, cosmos, and with nonhuman kin, an ideology which is regarded as “irrational” and “unreasonable” in contemporary research practices [12, 31, 32].

Secondly, the KTA model privileges primary research, which creates and sustains epistemic authority; the goal of knowledge development and synthesis occurs through systematic reviews, meta-synthesis and meta-analysis, which creates a dominant discourse that elevates primary research as having superiority over forms of knowledge development, thereby marginalizing approaches that do not align with Western empiricism.

Finally, the KTA cycle and the CIHR definition within Western academic contexts pay little if any attention to commitments to social justice and equity that are critical to any KT efforts needed to “improve the health of Canadians, provide more effective health services and products and strengthen the health care system”. KTA approaches grounded in Western Eurocentrism dominate and essentially erase knowledge and inquiry paradigms reflecting, for example, Black feminist ways of thinking and Indigenous cosmologies and epistemologies which view *knowledge* and *known* as interconnected [30, 33, 34] to advance social justice and equity that necessitate epistemological pluralism and ways of knowing beyond the empirical. KTA has been argued to be a critical role for advancing health equity to reduce and eliminate health inequalities within nursing and healthcare [35, 36]. In Canada, McMaster Health Forum has made great strides in strengthening health and social systems and demonstrates how working closely with individuals and organizations can contribute to a collaborative effort that addresses critical health and social system challenges [37]. However, KTA that inadequately theorizes social justice and equity goals runs the risk of perpetuating knowledge hierarchies that, ultimately, do not fully contribute to overall better health and strengthened healthcare systems. We now turn to discuss how Black feminist theorizing and Indigenous scholarship can provide direction for critical analysis of power, positionality, and hierarchy in research and KT that contributes to optimal impact and uptake in health policy and practice.

### The academy and power

The academy is inherently connected to colonial and gendered power and maintains an uncontested position at the top of the self-created hierarchy, wherein the academy rigidly defines epistemology and prescribes what counts are reliable, truthful, and valuable ways of knowing. These rigid and narrow approaches to knowledge are linked to cognitive imperialism, a form of cognitive manipulation used to repudiate other knowledge bases and values [7] and directly linked to settler architecture used to justify the advancement of Western rationalization. Built on the back of the imperialist world-making project, which was concerned with the material restructuring of the world that privileged whiteness and Eurocentric ways of being and knowing [13], the academy has promoted (and in some instances continues to promote) Western rationalization and legitimacy as universal common sense. This project of material conquest was tethered to the notion of “progress” as the signifier to assert “a sense of innate superiority and an overabundance of desire to bring progress” [5]. The assumed superiority of progress, and its connection to cognitive imperialism, has been central to the settler colonial project in Canada [29, 38]. Thus, the term “research” is inextricably linked to European imperialism and colonialism [5] and is bound up in the historical and contemporary positioning of those who deviate from the “norm” based on sex, gender, race, class, and/or ability as inferior or undesirable. Over time, the academy has recognized and acknowledged these instances of power dynamics and has employed processes to avoid such subjugation and exploitation of knowledge partners primarily through behavioural and human subjects research ethics boards (REBs) [39].

Although the creation of ethics review boards was to ensure protective practice for research participants throughout academia, several points of concern regarding power dynamics exist. RREBs act as gatekeepers of what knowledge is considered acceptable to “produce”, within the framework of ethical requirements. There is little questioning of potential biases in the assessment process and how the “value” of the anticipated knowledge is determined. Additionally, membership on REBs continues to privilege a particular population for decision-making; mainly, members of academic institutions with the requirement of one community member [39]. Rarely, if ever, community representation within specific studies is included. While these processes are intended for ethics surveillance within academic institutions, research ethics approval processes also reinforce power and control over knowledge partners. Furthermore, ethics regulations and guidelines are written by researchers for researchers and research participants in a protective paternalistic manner, reminiscent of colonial ideology, not in an intentional mutual process with knowledge partners [40]. Unsurprisingly, many if not all knowledge partners do not know their rights under these regulations and do not know the process of research or how it is constructed [40]. A consequence of REBs is the continuation of the imbalance of power that privileges researchers and disadvantages knowledge partners. Specifically, REBs are typically composed of faculty members within the same institution and can be insufficient to tackle the ethics of epistemologies of communities and partners that differ from the institutions underlying epistemological assumptions. In order to overcome hegemonic discourse, it is integral for research to take on an integrated approach that embraces KT from the very beginning of the research process. Such an approach can be achieved through IKT.

## Integrated knowledge translation

Integrated KT is a collaboration between researchers and knowledge partners that recognizes the expertise that both bring to the process [15]. Graham [17] describes the collaborative engagement of research partners in all stages as the bedrock of information sharing; refining the research problem; deciding on the most appropriate methodology/design, data collection, analysis; and disseminating the research findings. Integrated KT begins with the local context as its foundation for knowledge creation and continues with the development of contextually applicable interventions that are meaningful to the knowledge partner [41] As a model that promotes collaboration, IKT promotes an increase in resultant transformation in-group identity permitting added values in social and organizational perspectives [41]. The IKT model also creates space that enables a rebalancing of the expertise of any particular group engaged in research, thereby avoiding dominance of any partners.

Other concepts have been used to describe the IKT approach, such as participatory research, collaborative research, co-production of knowledge, action research, and engaged scholarship, to mention a few [42]. Broadly speaking, IKT and these related concepts continue to evolve as partners, researchers, policy-makers, and decision-makers continuously look for innovative ways of building knowledge to effect needed changes in healthcare [41]. The desired changes in healthcare are the ones enacted with the inclusion of knowledge partners as integral parts of the knowledge-building process [41].

## Expanding the epistemological ground

We argue that IKT holds considerable promise for promoting health equity in research, while also emphasizing multiples ways of knowing critical for understanding human experiences relevant for health policy and health services research and fostering capacity-building in research and practice. Researchers and *knowledge partners* recognize the value and expertise each brings to the process, contributing to the goal of minimizing power differentials and creating shared responsibility and understanding [2, 8, 15, 38].

We consider IKT as an umbrella term for other forms of integrated knowledge translation frameworks such as the co-creating KT (*CO-KT*), a process that synthesizes community-specific context outputs between researchers and knowledge users and draws on action and participatory research [43]; the *collaborative model*—shared reciprocity and accountability between researchers and knowledge users by breaking down typical role barriers in the research process [44]; and the *collaboraKTion framework*—engaging communities to refine, co-create, implement, and evaluate the effect of KT that is specific and sensitive to the context in which the new knowledge is generated and utilized [43, 45]. The above frameworks stress partnership and collaboration throughout the KT process and negotiation of shared power, exampling the IKT mandate of collaborative engagement of all partners from the beginning.

While advocating for IKT as an ideal approach to promote equity and power-sharing in the development of knowledge for relevant research evidence uptake in health policy and health services research, we recognize the inherent diversity in approaches and applications. Despite emerging evidence of IKT’s role in influencing research uptake and outcomes, it is neither well understood nor widely practised. Reasons such as the lack of clarity about how transformation occurs in the research process are lacking within literature on IKT designs [41]. Several studies state that IKT has been used, but do not adequately address the inherent power dynamics within the IKT process, nor are strategies to address power issues being published. Within the IKT discourse and practice, there are gaps, variations, and uncertainties that require clarification, such as conceptualization of the research question and process; contributions of partner engagement; issues of tokenism, scepticism, disciplinary norms; variation in language; inconsistencies with how low partner engagement is understood; and dialogue and inquiry to address gaps and overlaps [15, 46]. The underlying mandate of IKT, despite these limitations, has the potential to better align to advance the goals of KTA; the missing discourse within the current literature that is necessary for research aimed at promoting health equity and social justice is analyses of power in IKT.

### Where is the discourse on power and positionality in IKT?

Theorizing IKT in the literature requires its own critical self-reflection, as adequate accounting for and analysis of power has yet to be undertaken. Integrated KT has become the favoured approach to KT because of its assumed benefits for doing community-based participatory research (CBPR) and community-engaged studies. The application of IKT has been consistent throughout the literature as a framework that aligns with CBPR and community-engaged research processes, as these approaches make space for integrating the KT discussion from the very beginning [15, 41, 44, 45, 47]. While IKT has been acknowledged for its potential to advance more inclusive and diverse forms of KT, the inherent power dynamics associated with IKT are not explicitly identified in the literature, and discussion of the implication is insufficient. We argue that critical exploration of the power dynamics associated with IKT is required to advance KT scholarship.

Within IKT approaches, an assumption exists that “integration” of KT at the beginning of a study alone is sufficient to address power imbalances; however, without critical theorizing of positionality and standpoints, these frameworks can also reify hegemonic logics. Integration can easily become a “stir-and-mix” approach wherein the presence and recognition of “others” become the place marker for integration. This is not transformative change, but rather nicer packaging for the Eurocentric and Western knowledge paradigms status quo. For the purpose of explicating factors that constitute critical standpoints that can analyse power dynamics within IKT, we have identified four important points to consider: positions of power, rights, responsibilities, and relationality.

### Positions of power

At the core of power dynamics, we consider positions of power, where power is the capacity or potential to influence and affect the actions of others. We consider here positional power [48, 49] and personal power [50]. Positional power is the authority that a position is believed to confer upon a person in the organization’s structure and hierarchy, which is often influenced by “norms” and values [48, 49]. On the other hand, personal power is an individual’s skill and ability to influence people and events whether or not they have any formal authority [50]. These two points are important factors to consider when exploring power dynamics between researcher and knowledge partner. A researcher needs to recognize their position within the academy that prioritizes Western knowledge and knowledge production methods. This also includes the researcher being “expert” in the knowledge of academic processes and power structures. Therefore, the researcher must consider how their inherent position of power attempts to influence and control knowledge through their control over funding resources and the allocation of these resources. In contrast, knowledge partners should also understand how their positions of power relate to the research process. Take for instance the pre-established relationships and power structures within their own groups or communities. These could have beneficial outcomes or barriers to the research process, depending on their personal power or positional power, respectively. Knowledge partners also bring context-specific knowledge to the research process, despite having a lack of power secondary to inexperience with research processes. Therefore, knowledge partners as knowledge-keepers have an increased ability to address the problems at hand. This is important to acknowledge as it directly relates to the rights of knowledge partners.

### Rights

We understand rights as socially constructed, framed by rules, and related to a context [51]. In Canada, the *Canadian Charter of Rights and Freedoms* [52] of 1982 guarantees broad equality rights and other fundamental rights such as freedom of expression, freedom of assembly, and freedom of religion. However, given the history of “slash-and-grab” research [5], the rights of knowledge partners in relation to the rights of researchers is of critical concern, signalling the more extensive history and ongoing colonial practices often used to justify the dispossession of peoples situated at the margins. In the current Canadian context, with dedicated resources and attention given to advancing research projects for peoples situated at the margins, it is imperative to clearly define the rights of knowledge partners in the research process, which includes early consultation and involvement in the research process. Knowledge partners should also be primary decision-makers in *what* and *how* knowledge and questions of research benefit. Knowledge partners also require the right to access resources, education, and training to enable equitable contribution and partnership. Sharing responsibilities for the integration of the *many ways of knowing* into the KT process can address the inherent barriers, such as knowledge hierarchies, in the creation and analysis of data.

### Responsibilities

Responsibility within the research context is a state of being accountable to oneself and others by preventing the exploitation of knowledge partners, particularly when working with communities who are traditionally marginalized [53, 54]. The foundation of responsibility is contingent on the web of human relationships that constitute relationality between self, people, and spaces [55, 56]. Upholding the rights of the researcher and knowledge partners requires consideration of specific responsibilities that include a strength-oriented lens, an openness to questioning the research process and priorities, and clarifying group protocols. These negotiations of responsibilities are imbued with power; focus on how power is influencing the research and the collective responsibility between researcher and knowledge partners. These responsibilities vary depending on the nature of a person’s position, work, or function [57]. Researchers’ responsibilities are typically tied to resources, which include training, education, and other monetary costs [14, 35]. Researchers and knowledge partners may together negotiate their shared and distinct responsibilities for the uptake of research findings to their greatest impact to address the research problem under study.

### Relationality

Adequately theorizing power and upholding rights and responsibilities in inherently relational: reflexive relations with self (as the researcher), individuals and communities, and knowledge partners or stakeholders. Relationality has been theorized in nursing as founded on respect, trust, and mutuality [58], and these ways of being are relevant and critical when negotiating collaborative and IKT approaches. Decolonial conceptualizations of relationships have been based on reciprocity, respect, responsibility, and accountability [53]. Relationality is the means of connecting with self, other people, all living things including the land and past and future generations, and with knowledge [59]. It holds value in the reciprocal and accountable relationships formed with each of the aforementioned aspects. Relationality can be understood as transparent acts of sharing and connecting that honour reciprocity and require the mental, emotional, cognitive, and sometimes spiritual give and take from each party in the relationship. Due to the value placed on individuality in Western cultures and neoliberal values, relationality is often discouraged, and instead researchers are often encouraged to distance self from “participants”. Taking a relational approach to KT values collectivity opposes individualistic approaches and unseats neoliberal values, challenging fundamental positions of power traditionally bestowed upon and taken up by many academic researchers.

## Strategies to support the IKT process in addressing power dynamics

We now turn to discussing strategies to support the IKT process through critical analysis of power and positionality within the research context. These strategies include power*-sharing, dialogic communications, respect, collaboration, mutual learning, trust, transparency*, and *equity.*

Tackling power dynamics within the IKT process begins with the democratization of the process and disruption of the power hierarchy that often characterizes academic Western-centric research practices. This can be achieved through power-sharing [20], in which both researchers and partners recognize each other’s expertise and are flexible and open to practices that may differ from their own. Further, partners can be hired as coordinators of the research to increase their confidence and authority in the research process [60]. In turn, they can be best positioned to lead the dissemination of findings within their communities and the research context. Communities’ rights and interests should be recognized as articulated through OCAP [61] principles of ownership, control, access, and possession.

Power-sharing requires dialogue, an existential necessity for those who strive for transformative action [62]. A commitment to democratic research requires dialogic communication in the IKT process. Dialogic communication requires humility [62] and meaningful communication about the nature of the IKT process. Both researchers and partners ought to strive for transparent communications and conflict management strategies that are agreed upon by a communally developed framework [56]. Consensus should also be created from the outset regarding role definitions, the research agenda, research outcomes, and dissemination and ownership of data and findings. In turn, this posits that the objectives and needs of the research project are jointly identified by an integrated team of researchers and knowledge partners. These integrated teams should be cognizant of the technical know-how of the researchers and the researchers’ responsibility to explain concepts and jargon in non-technical and non-intimidating ways, as well as answering questions from partners without judging them as unnecessary.

Respect for diverse skills and knowledge brought to research is another fundamental principle for power-sharing to occur [53, 63], requiring equitable opportunities for participation without “gatekeeping” by those who hold academic positions within the project. For instance, collaboratively creating meeting agendas to ensure space is made for all voices can reflect the valuing of diverse perspectives, knowledge, and perspectives relevant to the study; and, rotating chairing of meetings between researchers and partners can inculcate the spirit of inclusiveness and self-worth, and foster collaboration.

Addressing power dynamics within the IKT process also demands collaborative ways of thinking and practising [20]. These collaborative practices can be achieved through time and investment from both parties in nurturing these relationships. Researchers can organize informal meetings, attend community hall meetings, committees, or volunteer in communities before approaching partners with a research plan [60]. The time and effort that researchers place in establishing collaborative partnerships can be fruitful when tackling expected and unexpected barriers. Collaboration can also promote a mutual learning environment in which researchers learn other ways of knowing in addition to their scientific knowledge and methodologies [63].

To achieve higher levels of collaboration, both researchers and partners need to establish trust, be respectful, and accept one another’s cultural context [40]. Transparent relationships can dissolve the artificial binary between researchers and knowledge partners, acknowledging that all are living as part of one community aiming to address the focus on the study.

### Critical reflexivity

Power and positionality are inherent with all research studies; yet we argue that a critically reflexive approach to IKT can better realize a critical analysis of power dynamics, language use, and Eurocentrism in the academy. Reflexivity is a “method that fully embraces and exploits the subjectivity of research” [64]. It is the active acknowledgment by the researcher that their own actions and decisions will inevitably impact the interpretation and context of the research inquiry [65]. Reflexivity is more than reflection; it requires a critical consciousness-raising and action derived from the researcher’s positionality and understanding of the study [55]. Understanding the researcher’s position within the study allows for insights into various power relationships that include intersectional systems of power such as racism, ageism, ableism, classism, and so forth [64]. Reflexivity also shifts the researcher’s understanding of the data by considering the ontological, epistemological, and axiological components of the self, intersubjectivity, and the colonization of knowledge [65]. Critical reflexivity provides a platform to pursue critical reflection not only on one’s self, but also to examine interpersonal relationships with partners, and examine health systems [55]. These three layers of reflexive practice allow for a broader understanding of positioning, power, and unequal relationships, enabling silenced voices to be heard whilst tracking the reciprocal working of power, including the researcher’s changing position [55] within the IKT process.

## Conclusion

Power differentials will never be completely erased in research partnerships. Yet, it is necessary to be cognizant of power dynamics and take adequate steps to minimize the disproportionate level at which they co-exist and their impact on the knowledge partner relationship and participation. There have been great strides in KT scholarship that have made a series of advancements, global and local, to promote collaboration within the complex reality of social problems and politics. However, the issues of power and positionality in research relationships and strategies to equalize relationships remain largely unaddressed. The strategies recommended in this paper will encourage equity in power, responsibility, rights, and process, while recognizing that power-sharing is flexible and adaptable to context and to the individuals/groups that are partnering. Within this space, power can produce knowledge [66] if it acknowledges the complexities of the researcher’s and knowledge partners’ positionality to focus on how *ways of being* come together in the creation of knowledge. The epistemological and ontological underpinnings of relationality and critical reflexivity underscore the importance of *being* in the knowledge partner relationship, thereby creating the foundation for moving IKT forward in health policy and health services research.

## Data Availability

Not applicable.
